# Coordinated cpSRP43 and cpSRP54 Abundance Is Essential for Tetrapyrrole Biosynthesis While cpSRP43 Is Independent of Retrograde Signaling

**DOI:** 10.3390/plants14121745

**Published:** 2025-06-06

**Authors:** Shuiling Ji, Huijiao Yao, Bernhard Grimm

**Affiliations:** 1 Key Laboratory of Pesticide & Chemical Biology of Ministry of Education, Hubei Key Laboratory of Genetic Regulation and Integrative Biology, School of Life Sciences, Central China Normal University, Wuhan 430079, China; 2 Institute of Biology/Plant Physiology, Humboldt-Universität zu Berlin, Philippstr.13, Building 12, 10099 Berlin, Germany

**Keywords:** chloroplast signal recognition particle (cpSRP), cpSRP43, cpSRP54, genomes-uncoupled (GUN), retrograde signaling, tetrapyrrole biosynthesis, protein abundance

## Abstract

The chloroplast signal recognition particle (cpSRP) components cpSRP43 and cpSRP54 not only form a complex with light-harvesting chlorophyll (Chl)-binding proteins to direct them to the thylakoid membrane, but also serve other functions. cpSRP43 independently acts as a chaperone for some tetrapyrrole biosynthesis (TBS) enzymes, while cpSRP54 participates in the co-translational targeting of plastid-encoded proteins. However, it remains unclear to what extent the two cpSRP components are coregulated—despite their distinct functions—and whether both participate in genomes-uncoupled (GUN)-mediated retrograde signaling. Here, we demonstrate that cpSRP43 and cpSRP54 accumulation is strongly interdependently controlled: overexpression of one protein increases the level of the other, while a deficiency in one of the two proteins leads to a simultaneous decrease in the other component. Disruption of this balance, e.g., by combining the overexpression of one component with a knockout of the other, results in severe chlorosis, stunted growth, and reduced levels of Chl and tetrapyrrole intermediates. Moreover, cpSRP43 deficiency exacerbates the pale-green phenotype of *gun4* and *gun5* mutants, highlighting a synergistic impact on TBS; however, cpSRP43 overexpression fails to rescue these defects. Remarkably, loss of cpSRP43 does not affect the expression of nuclear-encoded photosynthetic genes under intrinsic plastid stress, clearly demonstrating that cpSRP43 is not involved in plastid-to-nucleus retrograde signaling. Overall, our findings underscore that the fine-tuned expression of cpSRP43 and cpSRP54 is crucial for proper chloroplast function and pigment biosynthesis, while cpSRP43 alone does not participate in the retrograde signaling pathway.

## 1. Introduction

For many years now, the chloroplast signal recognition particle (cpSRP) pathway has been dedicated to the post-translational transfer of light-harvesting chlorophyll (Chl) a/b-binding proteins (LHCPs) from the stroma to the thylakoid membrane. Unlike canonical bacterial and cytosolic SRP pathways, which rely on an SRP RNA, the cpSRP system is uniquely composed of two protein subunits: a 54-kDa GTPase (cpSRP54) and a chloroplast-specific 43-kDa protein (cpSRP43) [1,2,3]. cpSRP43 plays a central role by binding directly to a conserved 18-residue hydrophobic motif (L18) within LHCP [4,5]. This interaction, that simultaneously conceals and transports LHCP, not only prevents the aggregation of LHCPs, but also primes them for delivery to the thylakoid membrane. This is achieved, when cpSRP54 provides the essential GTP-driven targeting signal through its interaction with the SRP receptor cpFtsY, which directs the complex to the thylakoid membrane via its conserved NG (N-terminal plus GTPase) domain [6,7]. At the thylakoid, cpSRP43 interacts via its chromodomains with a linear motif in the C-terminal region of the translocase Alb3, thereby ensuring the efficient docking of the LHCP-bound complex [8,9]. The cooperative action of cpSRP43 and cpSRP54 is ultimately finely regulated: cpSRP43 undergoes conformational changes upon assembling with cpSRP54 versus Alb3, ensuring LHCP is stably bound in the stroma and efficiently released at the membrane [10]. The cpSRP pathway is thus an example of a dynamic interplay between the function of a chaperone-subunit (cpSRP43) and the GTPase-subunit (cpSRP54) to achieve precise protein targeting.

While it was originally assumed that cpSRP54 and cpSRP43 act exclusively as obligatory heterodimers, more recent studies have shown that each subunit is also involved in different functions within the chloroplast. As the majority of cpSRP54 exists in a soluble form, less than half of cpSRP54 is associated with ribosomes and mediates the co-translational insertion of several chloroplast-encoded thylakoid proteins [11,12]. Ribosome profiling has demonstrated that the conserved C-terminal tail of cpSRP54 binds directly to ribosomal protein uL4 within the exit tunnel, thereby recruiting the nascent chain to the membrane translocon [12]. In addition, cpSRP54 and FtsH2 protease coordinate the proteostasis associated at the thylakoid membrane in *Arabidopsis* [13]. This multifunctional role of cpSRP54 underscores the versatility and complexity of the cpSRP system. So far, there is no convincing indication supporting the presence of a substantial free cpSRP54 pool in the stroma. Thus, cpSRP54, which is not associated with ribosomes, could predominantly be found to be complexed with cpSRP43.

Furthermore, the sole cpSRP43 has been initially shown to interact directly with glutamyl-tRNA reductase (GluTR), the enzyme catalyzing the first committed step in tetrapyrrole biosynthesis, thereby preventing its aggregation and enhancing the production of 5-aminolevulinate (ALA), the rate-limiting step for Chl and heme synthesis [14]. Intriguingly, when cpSRP43 is associated in the heterodimeric cpSRP43/cpSRP54 complex, its ability to stabilize tetrapyrrole biosynthetic enzymes is inhibited [15]. This suggests a regulatory mechanism by which cpSRP43 switches between a “transit complex” mode (bound to cpSRP54 to target LHCP) and a “chaperone” mode (free to interact with tetrapyrrole enzymes) [15]. Supporting this model, cpSRP54 itself appears to play a more limited role in chaperoning enzymes of Chl biosynthesis—for instance, cpSRP54 was shown to promote the thylakoid membrane association of the light-dependent protochlorophyllide oxidoreductase (LPOR) enzyme, whereas cpSRP43 primarily stabilizes LPOR in green leaves and during heat stress [16]. Thus, cpSRP43 and cpSRP54 can independently assist different clients in chloroplasts, and an appropriate balanced accumulation of both cpSRP components is likely critical for maintaining both efficient LHCP transport and a robust Chl biosynthetic capacity.

Chl biosynthesis, which relies particularly on a coordinated metabolite flux to avoid the accumulation of photodynamically active tetrapyrrole intermediates, also exhibits interdependencies for retrograde signaling from the plastid to the nucleus [17,18,19]. Chloroplasts convey their developmental and metabolic status to the nucleus to modulate the nuclear transcriptional control of photosynthesis-associated nuclear genes (PhANGs) [20]. Perturbations in tetrapyrrole metabolism have visualized this intracellular signaling. The well-described genomes-uncoupled (*GUN*) mutants of *Arabidopsis* have shown that defects in tetrapyrrole biosynthesis (TBS)—often caused by mutations in key components, such as GUN4 (a porphyrin-binding cofactor that stimulates magnesium (Mg) chelatase) and GUN5 (the CHLH subunit of Mg chelatase)—result in abnormal PhANG expression when chloroplast development is compromised [18,19]. The *gun4-1* allele harbors a point mutation in the GUN4 gene, causing a leucine-to-phenylalanine substitution at amino acid 88 (L88F) that destabilizes the protein and drastically reduces its abundance in the chloroplast. In contrast, the *gun4-3* allele contains a T-DNA insertion near the C-terminus of GUN4, leading to very low levels of a truncated, largely nonfunctional protein [19]. In *gun5-1*, a single nucleotide substitution (C→T) in the third exon converts an alanine to valine at position 990 (A990V) in the CHLH protein; this mutation, located in a highly conserved region, disrupts the CHLH function by weakening the Mg chelatase activity [18]. Collectively, these *GUN* mutants highlight distinct facets of how disrupted TBS in *Arabidopsis* impairs Chl production and disturbs plastid-to-nucleus signaling, underscoring the tight coupling of plastid pigment metabolism and nuclear gene expression.

Although the cpSRP pathway and plastid retrograde signaling have been separately studied in the past, recent evidence suggests a mutual interaction between these processes. cpSRP43 connects the LHCP insertion machinery with tetrapyrrole metabolism by chaperoning enzymes like GUN4 and GUN5/CHLH, raising the question of whether cpSRP43 might influence the activation of GUN-dependent signals. Moreover, from the perspective of protein homeostasis, the maintenance of an appropriate stoichiometry of cpSRP43 and cpSRP54 may be crucial. To date, the physiological consequences of deregulated cpSRP43/cpSRP54 levels in vivo remain poorly understood. Do the two genes for cpSRP43 or cpSRP54 show mutual transcriptional control and do both proteins require each other for stability and function in the chloroplast? And moreover, what happens to Chl biosynthesis, chloroplast development, and signaling when the balance of the expression of both cpSRP components is disrupted?

In this study, we address these critical questions by analyzing *Arabidopsis* lines with modified levels of cpSRP43 and cpSRP54. Our approach includes overexpression lines and single and double mutants that combine defects in the cpSRP pathway with mutations in the key components GUN4 or GUN5 of the GUN-mediated retrograde signaling pathway.

## 2. Results

### 2.1. Correlation of cpSRP43 and cpSRP54 Abundance in cpSRP54 Overexpression Lines

Although cpSRP43 and cpSRP54 form a heterodimeric cpSRP complex that facilitates the translocation of LHCPs across the chloroplast stroma to the thylakoid membrane, accumulating evidence indicates that additional pools of these proteins exist independently of the heterodimeric complex. Therefore, the question arose whether cpSRP43 and cpSRP54 abundances are strictly correlated.

**Figure 1:**
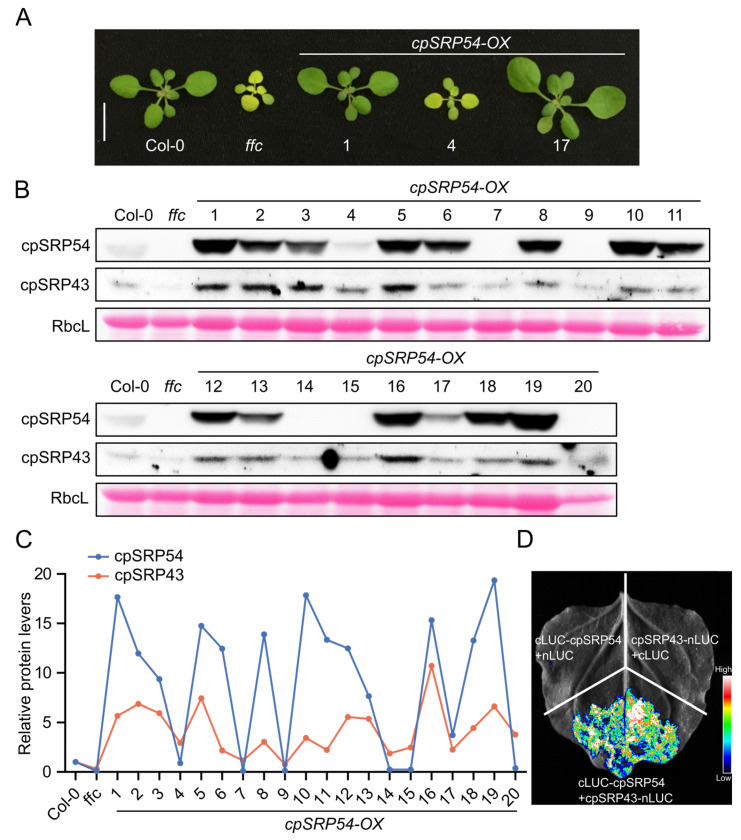
cpSRP43 abundance correlates with cpSRP54 abundance. (**A**) Representative images of the wild type (Col-0), the cpSRP54-deficient mutant (*ffc*), and three independent cpSRP54 overexpression lines (*cpSRP54-OX* #1, #4, and #17). Plants were grown under the standard conditions and photographs were taken at the same developmental stage. Scale bar = 1 cm. (**B**) Immunoblot analysis of cpSRP54 and cpSRP43 in Col-0, *ffc*, and a series of *cpSRP54-OX* lines (#1–20). RbcL (the large subunit of Rubisco) stained with Ponceau S serves as a loading control. (**C**) Relative protein levels of cpSRP54 and cpSRP43 in *ffc* and *cpSRP54-OX* lines compared with Col-0. The band intensities from (**B**) were quantified using ImageJ software (v1.8.0.345) and normalized to the corresponding RbcL signals. (**D**) Split Firefly Luciferase Complementation Imaging assay demonstrating the interaction between cpSRP54 and cpSRP43. *Nicotiana benthamiana* leaves were transiently co-expressed with cpSRP43 fused to the N-terminal fragment of firefly luciferase (nLUC) and cpSRP54 fused to the C-terminal fragment (cLUC). Negative controls included co-expression of cpSRP43-nLUC with cLUC alone, and cLUC-cpSRP54 with nLUC alone. Luminescence intensity is shown by the false color scale at the lower right, with higher intensity indicating stronger protein–protein interaction.

To address this issue, we generated a series of cpSRP54 overexpression lines (*cpSRP54-OX*) exhibiting varying elevated cpSRP54 protein levels. The overexpression of cpSRP54 in the cpSRP54-deficient mutant (*ffc*) significantly rescued the pale-green phenotype and restored the seedling morphology to resemble wild-type plants (Col-0) (Figure 1A). While *ffc* shows trace amounts of cpSRP43, line #4 (shown in Figure 1A) displayed extremely low levels of cpSRP54 and failed to rescue the pale-green phenotype. No cpSRP54 levels were immunologically detectable in lines #7, #9, #14, #15, and #20 and, therefore, these are not real *cpSRP54-OX* lines (Figure 1A,B). Immunoblot analyses showed that cpSRP43 abundance was closely correlated with cpSRP54 levels across the *cpSRP54-OX* lines: a higher accumulation of cpSRP54 corresponded to elevated cpSRP43 levels, whereas a reduced cpSRP54 content was associated with lower cpSRP43 abundance (Figure 1B). The quantitative analysis of the immunoblot signal further clarified this correlation, showing a synchronous increase and decrease in the cpSRP43 and cpSRP54 levels in different transgenic lines (Figure 1C). These results strongly suggest a strict correlation between cpSRP43 and cpSRP54 abundances, despite the presence of their distinct functional pools.

To further substantiate the physical interaction between cpSRP43 and cpSRP54, we performed a Split Firefly Luciferase Complementation Imaging (LCI) assay in *Nicotiana benthamiana* leaves. The co-expression of cpSRP43 fused to the N-terminal fragment of firefly luciferase (nLUC) and cpSRP54 fused to the C-terminal fragment (cLUC) resulted in a robust luminescence signal, confirming their direct interaction in vivo. In contrast, negative controls—consisting of cpSRP43-nLUC with cLUC alone or cLUC-cpSRP54 with nLUC alone—displayed only background luminescence (Figure 1D). These results, together with the abundance correlation analyses, provide both quantitative and direct biochemical evidence for the functional and physical association of cpSRP43 and cpSRP54.

### 2.2. Disruption of the Correlation Between cpSRP43 and cpSRP54 Abundance Impairs Plant Growth

**Figure 2:**
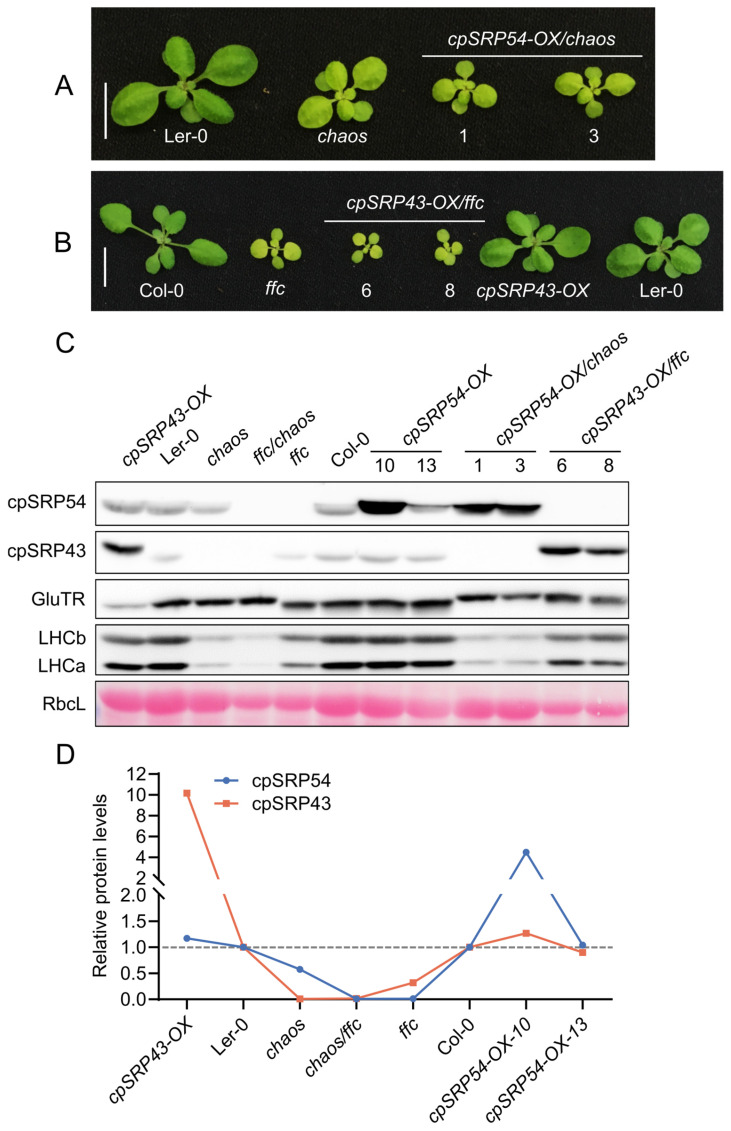
Disrupted cpSRP43–cpSRP54 balance impairs plant growth. (**A**) Representative images of Ler-0 (wild-type), the cpSRP43-deficient mutant *chaos*, and two independent cpSRP54 overexpression lines in the *chaos* background (*cpSRP54-OX*/*chaos* #1 and #3). Plants were grown under the same conditions and photographed at the same developmental stage. Scale bar = 1 cm. (**B**) Representative images of Col-0 (wild-type), the cpSRP54-deficient mutant *ffc*, two independent cpSRP43 overexpression lines in the *ffc* background (*cpSRP43-OX*/*ffc* #6 and #8), a *cpSRP43-OX* line in *chaos* background, and Ler-0. Scale bar = 1 cm. (**C**) Immunoblot analysis of cpSRP54, cpSRP43, GluTR (Glutamyl-tRNA reductase), LHCb (Light-harvesting chlorophyll a/b-binding protein b), and LHCa (Light-harvesting chlorophyll a/b-binding protein a) in the indicated genotypes. RbcL stained with Ponceau S serves as a loading control. (**D**) Quantification of relative cpSRP54 and cpSRP43 protein levels, normalized to their respective wild-type ecotype (Col-0 for *ffc* and *cpSRP54-OX* lines, Ler-0 for *chaos* and its derivative lines), based on the immunoblot signals from (**C**). The gray dashed line indicates the baseline levels of cpSRP54 and cpSRP43 in wild-type plants (Col-0 and Ler-0).

Given that cpSRP43 abundance is dependent on cpSRP54, we explored whether cpSRP54 levels are reciprocally affected by changes in cpSRP43 abundance. To this end, we used plant lines expressing wild-type levels (Ler-0), overexpressed levels (*cpSRP43-OX*), or no cpSRP43 (*chaos*). The phenotypic analysis revealed that *chaos* seedlings were pale green and exhibited growth retardation, whereas the overexpression of cpSRP43 in the wild type rescued these defects, yielding phenotypical seedlings comparable to those of Ler-0 (Figure 2A,B). Immunoblot analysis revealed that cpSRP54 levels progressively decreased from the *cpSRP43-OX* through Ler-0 to *chaos* (Figure 2C). The quantitative analysis confirmed this trend and clearly illustrates that the abundance of cpSRP54 declined in parallel with the decreasing cpSRP43 levels (Figure 2D). Conversely, in the series from the *ffc* to the *cpSRP54-OX*, cpSRP43 abundance consistently increased with elevated cpSRP54 levels (Figure 2C,D). These results indicate an interdependent correlation between the accumulation of cpSRP43 and cpSRP54.

To further investigate this relationship, we disrupted the correlation between cpSRP43 and cpSRP54 by generating double mutants: *ffc* was crossed with *cpSRP43-OX* and *chaos* was crossed with *cpSRP54-OX*, yielding the double mutants *cpSRP43-OX*/*ffc* and *cpSRP54-OX*/*chaos* (Figure 2A,B). It is noteworthy that the ecotype backgrounds differ in the lines used: *chaos* and *cpSRP43-OX* lines are Ler-0 seedlings, whereas *ffc* and *cpSRP54-OX* are Col-0 seedlings. We show the different electrophoretic mobility of GluTR of Ler-0 and Col-0 seedlings via immunoblotting to clarify the ecotype of the double mutants. Leaf extracts from both *cpSRP43-OX*/*ffc* and *cpSRP54-OX*/*chaos* showed GluTR mobility identical to those from Ler-0 (Figure 2C), confirming the genetic Ler-0 background of these double mutants.

The phenotypical analysis revealed that a genetic disruption of the abundance of cpSRP43 and cpSRP54 led to a strong impairment of seedling growth. Specifically, neither *cpSRP54-OX*/*chaos* nor *cpSRP43-OX*/*ffc* lines rescued the pale-green phenotype of the *chaos* or *ffc* mutants (Figure 2A,B). Instead, these double mutants exhibited significantly smaller and paler seedlings compared to their parental lines (Figure 2A,B), indicating that a genetic disruption of the correlative accumulation of cpSRP43 and cpSRP54 severely impairs plant growth. Despite this, the immunoblot profiles of GluTR, LHCb, and LHCa in the double mutants closely resembled those of the respective single null mutants (Figure 2C).

**Figure 3:**
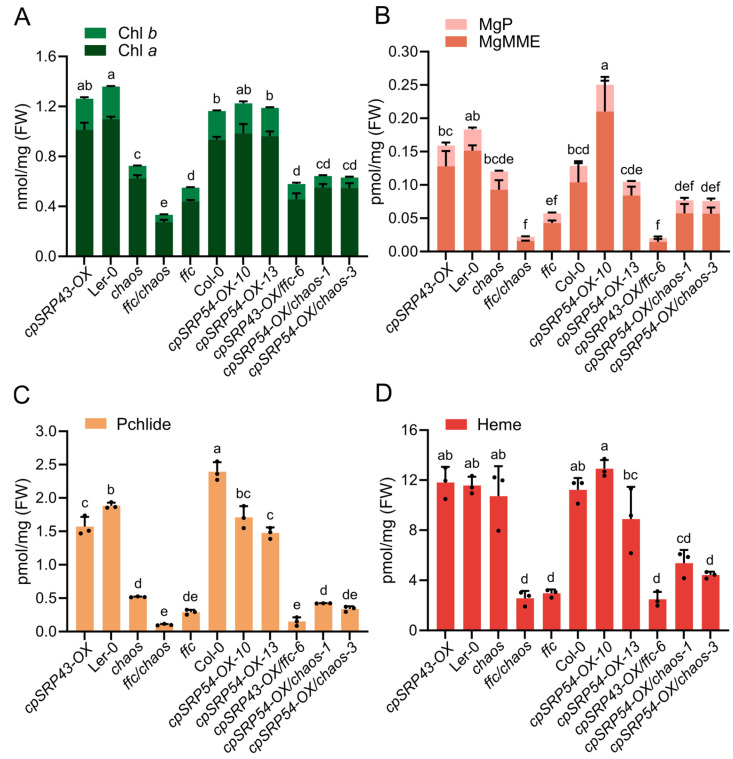
Disrupted cpSRP43–cpSRP54 balance impairs the tetrapyrrole biosynthesis pathway. (**A**) Quantification of chlorophyll *a* (Chl *a*) and chlorophyll *b* (Chl *b*) content in the indicated genotypes. (**B**) Analysis of tetrapyrrole intermediates, including Mg-protoporphyrin IX (MgP) and Mg-protoporphyrin IX monomethyl ester (MgMME) in the indicated genotypes. (**C**) Measurement of protochlorophyllide (Pchlide) levels across different *Arabidopsis* lines. (**D**) Heme content in the indicated genotypes. All data are presented as the mean ± SD from three biological replicates. Individual data points are represented by small black dots. Letters above the bars indicate significant differences, as determined by one-way ANOVA followed by Tukey’s HSD multiple comparison test (*p* < 0.05). FW, fresh weight.

Consistent with these phenotypes, the HPLC analyses showed significantly lower or comparable Chl contents in the double mutants relative to parental and wild-type lines (Figure 3A). The levels of tetrapyrrole intermediates Mg-protoporphyrin (MgP), Mg-protoporphyrin monomethyl ester (MgMME), and protochlorophyllide (Pchlide) were markedly reduced compared to their parental lines (Figure 3B,C). Correspondingly, the content of the end-product heme was also significantly lower in the double mutants compared with parental lines and the wild type (Figure 3D). Collectively, these findings strongly support that the maintenance of the correlated abundance of cpSRP43 and cpSRP54 is critical for normal chloroplast function, TBS, and overall plant growth.

### 2.3. Double Mutants for cpSRP43 and GUN4/GUN5 Are Severely Growth-Retarded and Impaired in Tetrapyrrole Biosynthesis

It has previously been shown that cpSRP43 acts as a chaperone, protecting GUN4 and GUN5 (CHLH) from their own aggregation under standard and heat shock conditions and thus preserving their stability [15]. However, it remains unclear whether the genetic relationship between cpSRP43 and GUN4/GUN5 is epistatic or synergistic. To address this question and clarify their common or mutual functional roles in chloroplast development and TBS, we investigated whether the simultaneous disruption of the expression of *cpSRP43* and *GUN4*/*GUN5* would exacerbate the defects observed in single mutants. Therefore, we generated double mutants by crossing *chaos* with *gun4-1*, *gun4-3*, and *gun5-1*.

**Figure 4:**
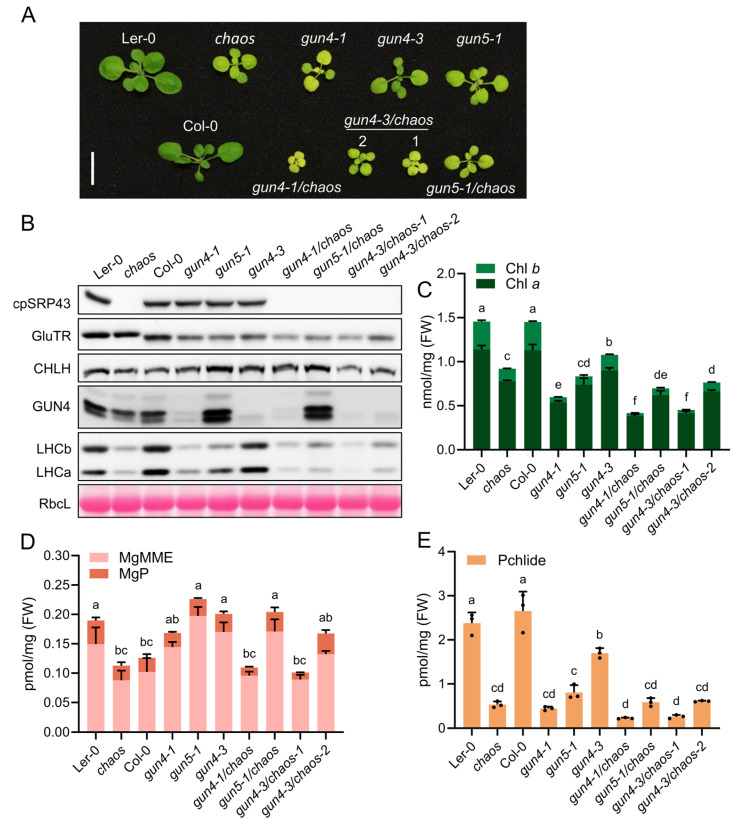
Combination of *chaos* and *gun* mutants exacerbates growth defects. (**A**) Representative phenotypes of wild-type (Ler-0 and Col-0), the *chaos* mutant, and *gun* mutants (*gun4-1*, *gun4-3*, and *gun5-1*), and double mutants (*gun4-1*/*chaos*, *gun5-1*/*chaos*, and *gun4-3*/*chaos* lines #1 and #2). Scale bar = 1 cm. (**B**) Immunoblot analysis of cpSRP43, GluTR, CHLH (H subunit of Mg-chelatase), GUN4 (genomes uncoupled 4), LHCb, and LHCa proteins across the indicated genotypes. RbcL stained with Ponceau S serves as a loading control. (**C**) Chl *a* and Chl *b* contents in the indicated genotypes. (**D**) Analysis of tetrapyrrole intermediates MgP and MgMME in the indicated genotypes. (**E**) Pchlide content in the indicated genotypes. All data are presented as the mean ± SD from three biological replicates. Individual data points are represented by small black dots. Letters above the bars indicate significant differences, as determined by one-way ANOVA followed by Tukey’s HSD multiple comparison test (*p* < 0.05). FW, fresh weight.

The phenotypic analyses showed that the resulting double mutants—*gun4-1*/*chaos*, *gun4-3*/*chaos*, and *gun5-1*/*chaos*—displayed severely impaired growth compared to their respective parental single mutants, as evidenced by significantly smaller and paler seedlings (Figure 4A). Consistent with these phenotypes, immunoblot analyses revealed substantial reductions in the abundance of exemplary proteins essential for TBS and the assembly of the photosynthetic complexes, such as GluTR, LHCb, and LHCa, in these double mutants (Figure 4B). Biochemical quantifications using HPLC showed that levels of Chl *a*/*b* were notably diminished in the double mutants relative to parental and wild-type lines (Figure 4C). Correspondingly, TBS intermediates, including MgP, MgMME, and Pchlide, exhibited significant decreases (Figure 4D,E). These results suggest that the simultaneous inactivation of cpSRP43 and GUN proteins involved in the Mg chelation of protoporphyrin IX exacerbates defects in TBS and chloroplast development, underscoring a critical genetic interaction between cpSRP43 function and GUN-mediated signaling as well as tetrapyrrole biosynthesis pathways that are all essential for maintenance of normal plant growth.

### 2.4. cpSRP43 Overexpression Does Not Rescue Gun Mutant Phenotypes

**Figure 5:**
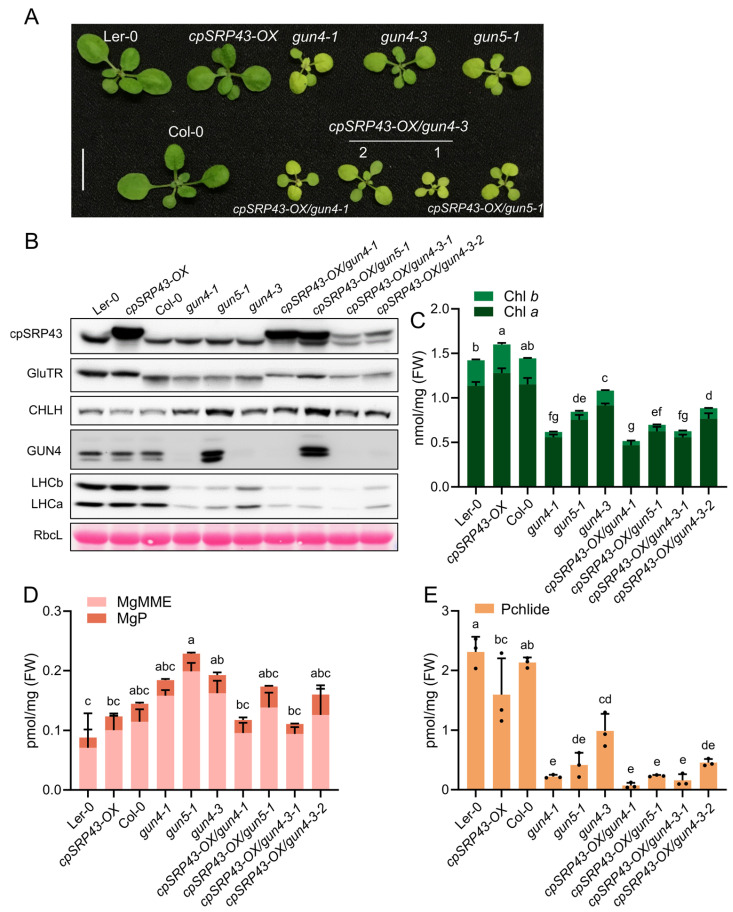
Overexpression of cpSRP43 cannot rescue the *gun* phenotype. (**A**) Representative phenotypes of wild-type (Ler-0 and Col-0), the *cpSRP43-OX* line, *gun* mutants (*gun4-1*, *gun4-3*, and *gun5-1*), and the corresponding double mutants (*cpSRP43-OX*/*gun4-1*, *cpSRP43-OX*/*gun4-3* lines #1 and #2, and *cpSRP43-OX*/*gun5-1*). Scale bar = 1 cm. (**B**) Immunoblot analysis of cpSRP43, GluTR, CHLH, GUN4, LHCb, and LHCa in the indicated genotypes. RbcL stained with Ponceau S serves as a loading control. (**C**) Quantification of Chl *a* and Chl *b* in the indicated genotypes. (**D**) Analysis of the tetrapyrrole intermediates MgP and MgMME in the indicated genotypes. (**E**) Quantification of Pchlide in the indicated genotypes. All data are presented as the mean ± SD from three biological replicates. Individual data points are represented by small black dots. Different letters above the bars indicate significant differences, as determined by one-way ANOVA followed by Tukey’s HSD multiple comparison test (*p* < 0.05). FW, fresh weight.

Given that the *chaos*/*gun* double mutants exhibit a more severe phenotype and indicate a synergistic relationship between the genes involved either in the cpSRP function and the TBS/retrograde signaling, we investigated whether cpSRP43 overexpression could alleviate the growth defects in the *gun4* and *gun5* mutants. We, therefore, compared the growth and leaf pigmentation of *cpSRP43-OX* lines in *gun4* and *gun5* backgrounds to their respective parental mutants (Figure 5A). The *cpSRP43-OX*/*gun* combination plants remained small and pale (chlorotic), closely resembling the stunted, light-green appearance of the *gun* mutants, underscoring that elevated cpSRP43 expression failed to rescue the characteristic pale and growth-deficient phenotype of *gun4* and *gun5* (Figure 5A).

Immunoblot analysis of key chloroplast proteins revealed significant changes in their abundance associated with the *gun* mutations (Figure 5B). For example, the protein levels of GluTR, CHLH, GUN4, LHCa, and LHCb were all altered in the *gun* mutant compared to the wild type, reflecting the disruptions in Chl biosynthesis, the formation of the photosynthetic complexes, and likely, retrograde signaling (Figure 5B). Notably, the *cpSRP43-OX*/*gun* showed protein abundance patterns similar to their respective *gun* parental lines for each marker protein examined. These results indicate that cpSRP43 overexpression does not compensate for the defects in chloroplast protein homeostasis caused by *gun* mutations. The expression of TBS enzymes (GluTR, CHLH, and GUN4) and photosystem antenna components (LHC proteins) in *cpSRP43-OX*/*gun* plants is consistent with the impaired functional properties of *gun* mutants (Figure 5B).

Consequently, *cpSRP43-OX*/*gun* accumulated similar amounts of Chl *a* and Chl *b* as the *gun* parental lines (Figure 5C). Likewise, the levels of MgMME, MgP, and Pchlide were generally comparable between *cpSRP43-OX*/*gun* lines and the corresponding *gun* mutants (Figure 5D,E). These data indicate that cpSRP43 overexpression does not substantially modify the core pigment biosynthetic performance in *gun* mutant plants. One notable exception was observed in the *gun4* background: the *cpSRP43-OX*/*gun4-3* line showed a further reduction in the total Chl content compared to the *gun4-3* mutant (Figure 5C), consistent with its slightly more pronounced pale leaf phenotype. Nevertheless, the pigment content of cpSRP43 overexpressors was basically the same as that of the *gun* mutants, emphasizing that cpSRP43 overexpression has little effect on TBS in the *gun4* or *gun5* background when Mg chelatase activity and Chl synthesis are reduced.

### 2.5. cpSRP43 Is Not Involved in Plastid-to-Nucleus Retrograde Signaling

**Figure 6:**
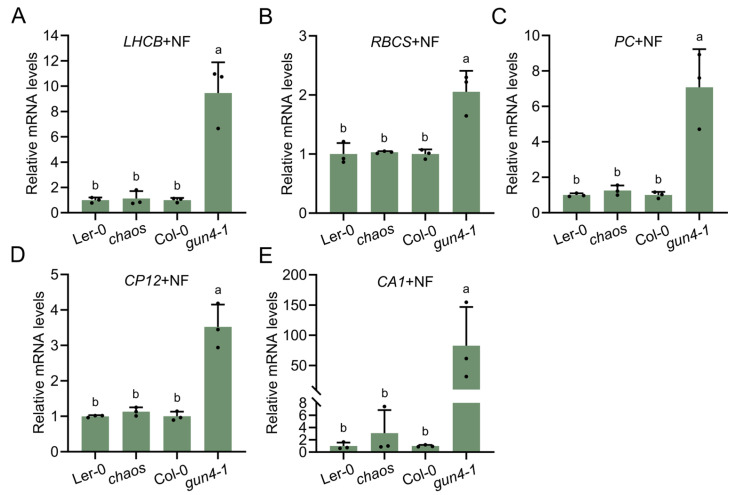
cpSRP43 is not involved in plastid-to-nucleus retrograde signaling pathway. (**A**–**E**) Relative mRNA levels of PhANGs, including *LIGHT HARVESTING CHLOROPHYLL-BINDING PROTEINS (LHCB)*, *RuBisCO SMALL SUBUNIT (RBCS)*, *PLASTOCYANIN (PC)*, *CHLOROPLAST PROTEIN 12 (CP12)*, and *CARBONIC ANHYDRASE1* (*CA1*). (**A**) *LHCB*, (**B**) *RBCS*, (**C**) *PC*, (**D**) *CP12*, and (**E**) *CA1* in Ler-0, the *chaos*, Col-0, and the *gun4-1* mutant treated with 5 μM norflurazon (NF). All data are presented as means ± SD from three biological replicates, with individual data points shown as small black dots. Letters above the bars indicate significant differences (*p* < 0.05) determined by one-way ANOVA followed by Tukey’s HSD multiple comparison test.

Molecular communication between plastids and the nucleus, known as retrograde signaling, plays a critical role in coordinating nuclear gene expression in response to the functional status of plastids. To explore whether cpSRP43 also contributes to plastid-localized retrograde signaling, we analyzed PhANG transcript levels in *chaos* under conditions that disrupt plastid biogenesis (e.g., NF treatment). As a positive control, the *gun4-1* mutant exhibited an elevated expression of all tested PhANGs compared to the wild type (Col-0) (Figure 6A–E). In contrast, the *chaos* mutant showed PhANG expression comparable to that of the wild type (Ler-0), suggesting that the absence of cpSRP43 does not disrupt plastid-to-nucleus retrograde signaling (Figure 6A–E).

The DMSO-treated group served as the control for the NF treatment and the results indicated that DMSO did not significantly affect PhANG expression in either *chaos* or *gun4-1* compared with the wild type (Figure S1). Collectively, these findings indicate that cpSRP43 is not involved in the plastid-to-nucleus retrograde signaling pathway, and makes it possible to distinguish its role from that of GUN4 and GUN5, which, in addition to their function in the Mg chelation of protoporphyrin, are assigned a role in retrograde signal transmission.

## 3. Discussion

### 3.1. Interdependence of cpSRP43 and cpSRP54 Abundance

Our findings reveal a strong functional interdependence between cpSRP43 and cpSRP54 in *Arabidopsis* and emphasize that these two cpSRP components perform closely related functions. This mutual dependence of the expression of both proteins is remarkable considering that cpSRP43 and cpSRP54 have other functions in addition to the role of the heterodimeric cpSRP complex for the LHCP transfer through the stroma. cpSRP43 not only partners with cpSRP54 to target LHCPs to the thylakoid membrane [3,21], but it also acts independently as a chaperone for enzymes of the TBS pathway [14,15,16]. Conversely, cpSRP54 has its own role in the co-translational targeting of certain chloroplast-encoded proteins in cooperation with other auxiliary factors [12]. Despite these separate activities, our results indicate an obvious balance in cpSRP43 and cpSRP54 accumulation in plant cells. This coordination likely serves to synchronize Chl production with the availability of LHCP apoproteins, thereby linking pigment biosynthesis to thylakoid protein insertion in a highly regulated fashion.

Consistent with a model of mutual stabilization, altering the level of one cpSRP subunit had a direct impact on the abundance of the other protein. In c*pSRP54-OX*, cpSRP43 protein levels rose in parallel with cpSRP54 levels, whereas in a cpSRP43-overexpressing line, cpSRP54 levels were elevated relative to the wild type. Conversely, the absence of either subunit led to a marked decrease in the steady-state level of its partner. These observations strongly suggest that cpSRP43 and cpSRP54 post-translationally stabilize each other, likely through the formation of a cpSRP43–cpSRP54 complex.

Disrupting the cpSRP43–cpSRP54 balance had severe consequences for plant growth and chloroplast development. Neither increasing cpSRP43 in a cpSRP54-null mutant nor increasing cpSRP54 in a cpSRP43-null background could rescue the characteristic pale, stunted phenotype of the single mutants. This outcome indicates that an excess of one subunit cannot compensate for the loss of its partner. On the contrary, such an imbalance seems to be even more harmful. One possible interpretation is that any unpaired cpSRP subunit might be nonfunctional and could even affect the role of other interacting proteins or could also affect the quality control for other functional protein groups. Meanwhile, the limiting subunit, especially cpSRP54, is insufficient to carry out the essential role of the cpSRP pathway in LHCP trafficking [3,22,23]. The pronounced growth impairment in these mutants underscores that both cpSRP43 and cpSRP54 are required in concert and that their functions in chloroplast protein targeting (and perhaps other processes) are not redundant.

### 3.2. Synergistic Defects in cpSRP and GUN4/GUN5 Double Mutants

A major outcome of our study is the severe phenotype observed when cpSRP mutations are combined with lesions in the *GUN4* or *GUN5* genes. Individually, *gun4* and *gun5* mutants are pale green and exhibit reduced Chl levels due to impaired Mg chelation within the biosynthetic pathway. However, when the *chaos* mutation is combined with *gun4* or *gun5*, the double mutants are dramatically smaller, more chlorotic, and accumulate even less Chl than either single mutant. This synergistic effect can be explained by considering how cpSRP and GUN4/GUN5 each contribute to the production and utilization of Chl in the photosynthetic apparatus via different mechanisms. GUN5/CHLH functions as an Mg chelatase subunit directly responsible for inserting Mg^2+^ into protoporphyrin IX, a critical step in Chl biosynthesis [18], while GUN4 acts as a cofactor that binds porphyrins, stimulates Mg-chelatase activity, and participates in sensing chloroplast tetrapyrrole levels for signaling [19,24,25]. Defects in either GUN4 or GUN5 result in less Chl availability for photosynthesis. In contrast, a cpSRP43 defect leads to Chl deficiency by preventing the proper insertion of LHCPs and by destabilizing enzymes such as CHLH and GUN4 themselves [15]. Essentially, a *cpSRP43* mutant simultaneously hampers the assembly of Chl-binding proteins and undermines the pigment biosynthesis, as evidenced by the reduced CHLH and GUN4 protein levels observed in *chaos*. The lack of pigments also destabilized the chlorophyll-binding proteins of the photosynthetic protein complexes [26,27]. The chloroplasts of double mutant *gun4*/*chaos* and *gun5*/*chaos,* therefore, experience a twofold deficit, which cannot be compensated. These deficiencies in the function of GUN4/5 and cpSRP components lead to an enhanced failure in Chl accumulation and ultimately to an arrest in chloroplast biogenesis (Figure 4). This amplified deficiency manifests itself in the extreme chlorosis and growth arrest that we have observed.

### 3.3. cpSRP43 Overexpression Cannot Rescue Gun Mutants

Our experiments showed that overexpressing cpSRP43 in *gun4* or *gun5* knock-down mutants failed to ameliorate their chlorotic, slow-growing phenotypes. This outcome is actually not surprising: the partial block of Mg-chelatase activity in these *gun* mutants impairs the tetrapyrrole metabolism, which apparently cannot be overcome by providing more of a cpSRP43 chaperone capacity. Regardless of how much cpSRP43 is present, it cannot directly increase the production of Mg chelatase or the synthesis of ALA, and thus the Chl precursor flow remains limited. It only chaperones existing subunits of Mg chelatase. For example, in wild-type plants, boosting cpSRP43 or cpSRP54 alone does not lead to higher Chl levels than normal, indicating that the Chl biosynthesis was already working at its maximal metabolic flow rate in the presence of normal cpSRP levels. cpSRP components are not a limiting factor for Chl synthesis, but only another supporting factor among several post-translational control mechanisms for this metabolic pathway [14,28]. In contrast, the overexpression of GUN4 can increase Chl production, as GUN4 directly enhances enzyme activities in the pathway [29]. Our *cpSRP43-OX*/*gun4* experiment reinforces this understanding: without GUN4, the rate-limiting steps of the Chl synthesis cannot be accelerated. These observations highlight the specificity of cpSRP43′s role—it is necessary as a chaperone for proper chloroplast biogenesis, but not sufficient to drive it when crucial biosynthetic components are missing. Any attempt to enhance the Chl content or photosystem assembly must, therefore, address the metabolic bottlenecks (like the Mg-chelation step) in addition to providing assembly factors and chaperones.

### 3.4. cpSRP43 and Retrograde Signaling

Despite the significant role of cpSRP43 in chloroplast development, our data clearly indicate that it does not participate in plastid-to-nucleus retrograde signaling (Figure 6). Mutations in *GUN4* or *GUN5* impair this signaling. *chaos* did not show a *gun*-type modified expression of PhANGs. When chloroplast development was chemically disrupted (by NF treatment), *chaos* seedlings repressed PhANG transcripts to wild-type levels, confirming no impact on the GUN-mediated retrograde signaling pathway. This finding allows a clear distinction between the roles of cpSRP43 and those of GUN4/GUN5; the latter influence both Chl synthesis and is involved in retrograde signaling, while cpSRP43 impacts Chl synthesis and utilization without affecting nuclear feedback signaling. The albino or pale phenotype of *chaos* mutants suggests multiple layers of the chloroplast dysfunction responses. Chloroplast assembly is compromised due to LHCP targeting failure and enzyme instability, but the mutants still produce proper retrograde signals to downregulate nuclear gene expression. Thus, cpSRP43 functions downstream of retrograde cue generation and influences the chloroplast assembly, but not the signaling mechanism itself.

### 3.5. Coordination of Chloroplast Biogenesis and Future Directions

Together, these results highlight how chloroplast biogenesis relies on the coordination of protein-targeting machineries with metabolic pathways. The cpSRP system and the tetrapyrrole biosynthetic pathway must work in tandem to achieve efficient Chl incorporation into photosynthetic complexes. An interruption in one activity is detrimental, but an interruption in both areas is disastrous, as we have seen. This interdependence exemplifies a broader principle of organelle development: the stoichiometry of multi-component processes (like pigment synthesis and apoprotein integration) must be tightly regulated to avoid imbalances that could lead to proteotoxic stress or metabolite accumulation. From an applied perspective, understanding this coordination could inform strategies to improve the plant vitality. For instance, balancing the expression of Chl-binding proteins with the rate of Chl production might be a key consideration in engineering crops with an enhanced photosynthetic performance. Future research directions include elucidating mechanisms maintaining cpSRP43/cpSRP54 balance, understanding adaptive regulation under stress conditions, and exploring interactions with other chloroplast assembly pathways and signaling networks. These studies could significantly enhance strategies to improve crop vitality and stress tolerance.

## 4. Materials and Methods

### 4.1. Plant Materials and Growth Conditions

*Arabidopsis thaliana* seedlings were grown in a 16 h light/8 h dark period on soil in a growth chamber (CLF PlantClimatics, Wertingen, Germany) at 22 °C and 100 µmol photons m^−2^s^−1^. Unless otherwise stated, the standard growth condition in this study is 16 h light/ 8 h dark, 100 µmol photons m^−2^s^−1^ at 22 °C. The information on the *Arabidopsis* lines used in this study is listed in Table S1.

### 4.2. Crossing of Arabidopsis Thaliana

*Arabidopsis* crosses were performed by emasculating unopened flower buds of designated maternal plants using fine forceps under a stereomicroscope (Olympus, Tokyo, Japan). Pollen from selected paternal plants was then applied directly onto the stigma of emasculated flowers. Crossed plants were marked and covered loosely to avoid contamination. Mature siliques were individually harvested, and resulting F1 seeds were collected, stratified at 4 °C for two days, and then germinated for subsequent analysis.

### 4.3. Analysis of TBS Intermediates and End-Products

Rosette leaves (40–60 mg) were harvested and their fresh weight (FW) was determined. TBS intermediates and end-products were extracted from frozen or lyophilized leaf powders in 400 μL of ice-cold pigment-extraction buffer (acetone: 0.2 M NH_4_OH = 9:1, *v*/*v*) at −20 °C for at least 1 h. After centrifugation (14,000× *g*, 20 min at 4 °C), the supernatant was subjected to HPLC. The pellet was retained for extraction of non-covalently bound heme using AHD buffer (acetone: hydrochloric acid: dimethyl sulfoxide = 10:0.5:2, *v*/*v*/*v*) at room temperature. HPLC analyses were conducted using the Agilent 1100 or 1290 HPLC system equipped with a diode array and fluorescence detectors (Agilent Technologies, Santa Clara, CA, USA), essentially as described previously [30].

### 4.4. Norflurazon Treatment

The sterile cultivation of *Arabidopsis* plants was conducted on Petri dishes with Murashige and Skoog medium (MS, with vitamins, Duchefa Biochemie, Haarlem, The Netherlands) mixed with plant cultivation agar (4.43 g/L MS, 0.5 g/L MES, 8 g/L agar, and pH 5.7 with NaOH). About 50 μL of seeds were incubated for 10 min in 1 mL of sterilization solution (70% ethanol (*v*/*v*), 0.05% (*v*/*v*) Triton X-100). The sterilization solution was then removed and the seeds were washed in 70% ethanol (*v*/*v*) twice. Then, the seeds were washed with 100% ethanol (*v*/*v*) twice and dried. The sterilized seeds were sown either dry or mixed with sterile water. To analyze the retrograde signaling pathway [17], *Arabidopsis* seeds were grown on the sterilized MS plates in the presence of 5 μM norflurazon (NF, 5 mM in DMSO as stock). After stratification for 2 days, seedlings were grown in continuous light for 5–6 days.

### 4.5. RNA Extraction and qRT-PCR

Total RNA was extracted from *Arabidopsis* leaves frozen in liquid nitrogen using the citric-acid extraction method [31]. Aliquots (2 μg) of DNase-treated RNA were reverse-transcribed using oligo (dT)_18_ primers and RevertAid reverse transcriptase (Thermo Fisher Scientific, Waltham, MA, USA). qPCR was carried out in the CFX96-C1000 96-well plate thermocycler (Bio-Rad, Hercules, CA, USA) by using 2×qPCR Mastermix (Bimake, Houston, TX, USA). *Actin* (At3g18780) was routinely used as the reference gene. Relative expression was calculated in Bio-Rad CFX Manager v1.6 using the 2^−ΔΔCt^ method. Primers for qRT-PCR are listed in Table S2.

### 4.6. Protein Extraction and Western Blot Analysis

Whole 14–18-day-old rosette seedlings were harvested from 3–6 individual plants of each genotype. In the case of plants grown under standard conditions, freshly harvested leaves were ground in liquid nitrogen and total leaf proteins were extracted from the powder using 2×Laemmli buffer [100 mM Tris-HCl pH 6.8, 4% (*w*/*v*) SDS, 20% (*v*/*v*) glycerol, 200 mM DTT, and 0.01% Bromophenol Blue] and incubated at 95 °C for 10 min. The protein concentration was determined and normalized to leaf fresh weight. In the case of heat shock experiments, rosette leaves were harvested before or after heat treatment and ground in liquid nitrogen. Total leaf protein was extracted from frozen plant material in PEB buffer [2% (*w*/*v*) SDS, 56 mM Na_2_CO_3_, 12% (*w*/*v*) sucrose, and 2 mM EDTA] and protein concentrations were determined using the Pierce BCA Protein Assay Kit (Thermo Fisher Scientific). All samples in PEB buffer were diluted to the same protein concentration, supplemented with 56 mM DTT, and incubated at 70 °C for 20 min. Aliquots (15 µg) of protein were subjected to SDS-PAGE (sodium dodecyl sulfate–polyacrylamide gel electrophoresis), transferred to nitrocellulose membranes (GE Healthcare, Chicago, IL, USA), and probed with specific antibodies. Antibodies against GluTR (1:1000), GUN4 (1:2000) were generated in our laboratory. Antibodies against cpSRP43 (1:2500) and cpSRP54 (1:2500) were kindly donated by Prof. Danja Schünemann (Ruhr University, Bochum, Germany). Those directed against CHLH (1:1000) were kindly provided by Dr. Da-Peng Zhang (Tsinghua University, Beijing, China). Immunoblotting signals visualized by Clarity^TM^ Western ECL (Bio-Rad) were detected with a CCD camera (Intas Science Imaging Instruments, Göttingen, Germany).

### 4.7. Split Firefly Luciferase Complementation Imaging Assay

The coding sequence (CDS) of cpSRP43, excluding the chloroplast transit peptide, was cloned into the JW771 vector (Bio-Vector NTCC, Beijing, China) to create a fusion with the N-terminal end of firefly luciferase (nLUC). Similarly, the CDS of cpSRP54 lacking its transit peptide was cloned into the JW772 vector (Bio-Vector NTCC, Beijing, China), resulting in a fusion with the C-terminal of firefly luciferase (cLUC). *Agrobacterium tumefaciens*-mediated infiltration was employed to co-express cpSRP43-nLUC and cLUC-cpSRP54 in *Nicotiana benthamiana* leaves, using the GV3101 strain (containing the helper plasmid pSoup-P19), following the procedure described by Gou et al. [32]. As negative controls, the empty JW771 and JW772 vectors were infiltrated individually. Luciferase activity was visualized 48–72 h after infiltration by spraying 0.8 mM D-luciferin (aladdin, Shanghai, China) and detecting chemiluminescence with a Tanon-5200 Multi imaging system (Tanon Science & Technology, Shanghai, China).

### 4.8. Image Processing and Graphic Evaluation

Image files (Western blots, protein gels, photographs, etc.) were processed with Photoshop CS3 (v10.0.1, Adobe, San Jose, CA, USA) and Inkscape (v1.2.1, https://inkscape.org/). Blot signals of proteins were quantified densitometrically using the ImageJ software (v1.8.0.345, NIH, Bethesda, Maryland, USA), with all quantifications performed using standardized protocols, including background subtraction, to minimize the potential subjective nature of this approach. Graphical representations were generated in GraphPad Prism 8.0 or v9.1.2 (226). Statistical analyses were performed using Excel 2016 or GraphPad Prism v.9.1.2 (226).

## 5. Conclusions

In summary, this study demonstrates that cpSRP43 and cpSRP54 function in a mutually dependent manner to support chloroplast development in *Arabidopsis thaliana*. A balanced abundance of these cpSRP subunits is essential for normal Chl biosynthesis and the proper assembly of light-harvesting complexes, as any disruption of their equilibrium leads to impaired growth and pale, chlorotic phenotypes. Moreover, although the cpSRP and the functions of the two proteins GUN5 and GUN4 for Chl synthesis and retrograde signaling synergistically influence TBS, our findings reveal that cpSRP43—despite its essential role in chloroplast protein and pigment homeostasis—does not participate in plastid-to-nucleus retrograde signaling. This distinction clearly sets its function apart from that of the GENOMES-UNCOUPLED regulators GUN4 and GUN5.

## Data Availability

All raw data and Supplementary Files supporting the findings of this study will be uploaded and made available alongside the manuscript. Further information or additional datasets are available from the corresponding author upon reasonable request.

## Supplementary Materials

The following supporting information can be downloaded at: https://www.mdpi.com/article/10.3390/plants14121745/s1, Figure S1: Expression of nuclear-encoded photosynthesis-associated genes (*PhANGs*) in *chaos* and *gun4-1* mutants under standard conditions (without NF); Table S1: Genotypes used and analyzed in this study; Table S2: List of primers used in this study and raw data.
