# Supplementary material for: Coordinated cpSRP43 and cpSRP54 Abundance Is Essential for Tetrapyrrole Biosynthesis While cpSRP43 Is Independent of Retrograde Signaling
Source: Plants (Basel). 2025 Jun 6;14(12):1745. doi: 10.3390/plants14121745 (PMC12197082; doi:10.3390/plants14121745)
Supplement: Supplementary file 1 [file plants-14-01745-s001.zip › plants-3656877-supplementary-final.pdf]

Supplementary file

Coordinated cpSRP43 and cpSRP54 Abundance Is Essential for Tetrapyrrole Biosynthesis While cpSRP43 Is Independent of Retrograde Signaling

Shuiling Ji [1,2,*] , Huijiao Yao [1] and Bernhard Grimm [2,*]

[1] Key Laboratory of Pesticide & Chemical Biology of Ministry of Education, Hubei Key Laboratory of Genetic Regulation and Integrative Biology, School of Life Sciences, Central China Normal University, Wuhan, China

[2] Institute of Biology/Plant Physiology, Humboldt-Universität zu Berlin, Philippstr.13, Building 12, 10099, Berlin, Germany

* Correspondence:

Shuiling Ji - shuiling.ji@ccnu.edu.cn

Bernhard Grimm - bernhard.grimm@rz.hu-berlin.de

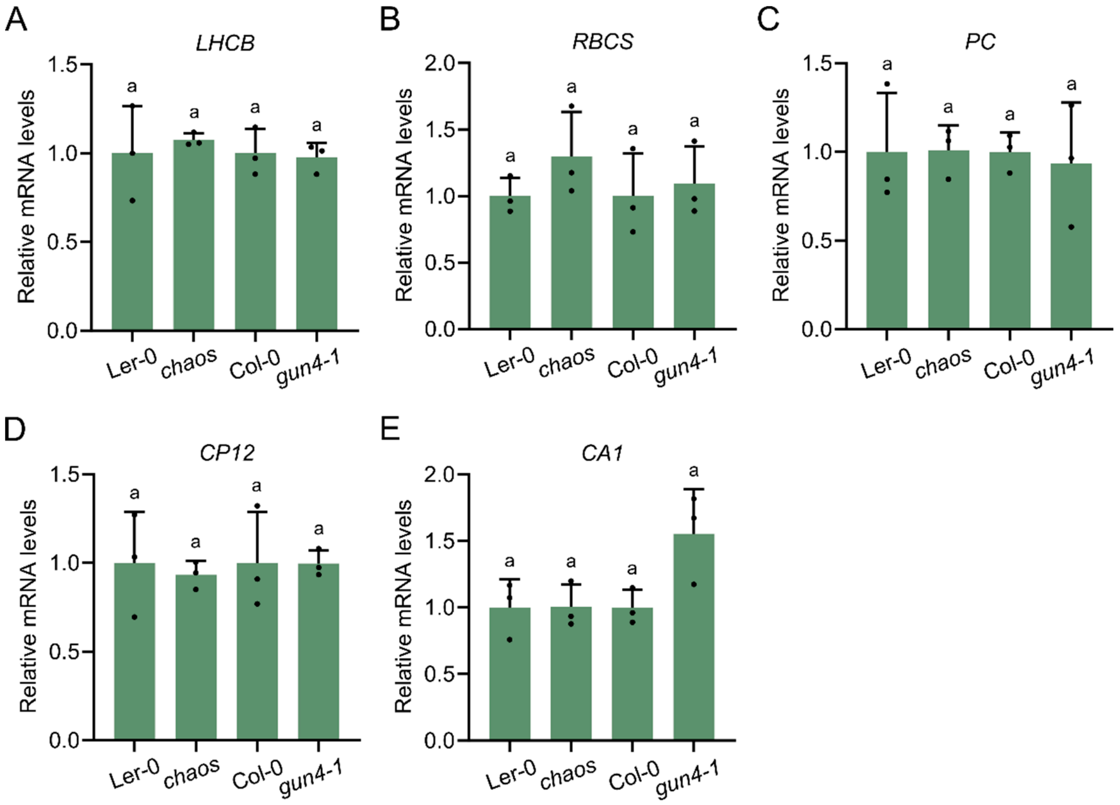

Figure S1. Expression of nuclear-encoded photosynthesis-associated genes (PhANGs) in *chaos* and *gun4-1* mutants under standard conditions (without NF). (**A–E**) Relative mRNA levels of (**A**) *LHCB*, (**B**) *RBCS*, (**C**) *PC*, (**D**) *CP12*, and (**E**) *CA1* in Ler-0, the *chaos* (cpSRP43-deficient) mutant, Col-0, and the *gun4-1* mutant. All data are presented as the mean ± SD from three biological replicates, with individual data points shown as small black dots. Letters above the bars indicate statistically significant differences ($P < 0.05$) determined by one-way ANOVA followed by Tukey's multiple comparisons test.

Table S1. Genotypes used and analyzed in this study

Line	Gene ID	Description	Source
Col-0	-	*Arabidopsis* Columbia wild type (WT)	-
Ler-0	-	*Arabidopsis* Landsberg erecta wild type (WT)	-
chaos	AT2G47450	*cpSRP43* knock-out mutant	[1]
cpSRP43-OX	AT2G47450	*chaos* complementation line expressing cpSRP43	[2]
ffc	AT1G15310	*cpSRP54* knock-out mutant	[3]
cpSRP54-OX	AT1G15310	*ffc* complementation line expressing cpSRP43	[4]
ffc/chaos	AT2G47450<br>AT1G15310	*cpSRP43* and *cpSRP54* double knock-out mutant	[3]
cpSRP54-OX/chaos	AT1G15310<br>AT2G47450	Overexpressing cpSRP54 in *chaos*	This study
cpSRP43-OX/ffc	AT2G47450<br>AT1G15310	Overexpressing cpSRP43 in *ffc*	This study
gun4-1	AT3G59400	Leu88->Phe8 mutation in GUN4	[5]
gun4-3	AT3G59400	T-DNA within the coding region of the C terminus in GUN4	[5]
gun5-1	AT5G13630	Ala990->Val990 mutation in CHLH/GUN5	[6]
gun4-1/chaos	AT3G59400<br>AT2G47450	*gun4-1* and *chaos* double mutant	This study

gun4-3/chaos	AT3G59400<br>AT2G47450	*gun4-3* and *chaos* double mutant	This study
gun5-1/chaos	AT5G13630<br>AT2G47450	*gun5-1* and *chaos* double mutant	This study

Table S2. List of primers used in this study

Name	Sequences (5’->3’)	Reference	Purpose
Actin2-Fw	CTTCCCTCAGCACATTCCAG	[7]	qPCR
Actin2-Rev	GACCTGCCTCATCATACTCG	[7]	qPCR
LHCB-Fw	GGACTTGCTTTACCCCGGTG	[7]	qPCR
LHCB-Rev	TCGGTAGCAAGACCCAATGG	[7]	qPCR
CA1- Fw	TGTGTCCATCACACGTTCTGG	[7]	qPCR
CA1- Rev	GGACCACGAAGGCATCTCCT	[7]	qPCR
RBCS-Fw	GCAACGGCGGAAGAGTTAAC	[7]	qPCR
RBCS-Rev	TCTTTCCAATCGGAGGCCA	[7]	qPCR
PC- Fw	TGGTGTTCGACGAAGACGAG	[7]	qPCR
pc- Rev	AGATCTTGCTTGCGTCCACA	[7]	qPCR
CP12- Fw	CGGACCCTTTGGAGGAATACTG	[7]	qPCR
CP12-Rev	GGCACTCGTTGGTCTCAGGA	[7]	qPCR
772-cpSRP54-Fw	GTACGCGTCCCGGGGCGGTACCCGGGATCCGGAGATGTTTGGTCAGTTGACTGGTG		LCI
772-cpSRP54-Rev	GATGATACGAACGAAAGCTCTGCAGGTCGACTTAGTTACCAGAGCCGAAGCCACGAG		LCI
771-	CACGGGGGACGAGCTCGGTACCCGGGATCCATGGCCGCC		LCI

cpSRP43-Fw	GTACAAAGAAACTACG		
771-cpSRP43-Rev	CGCCCCGGGACGCGTACGA GATCTGGTCGACTTCATTCA TTGGTTGTTGTTGTTGG		LCI

Raw data

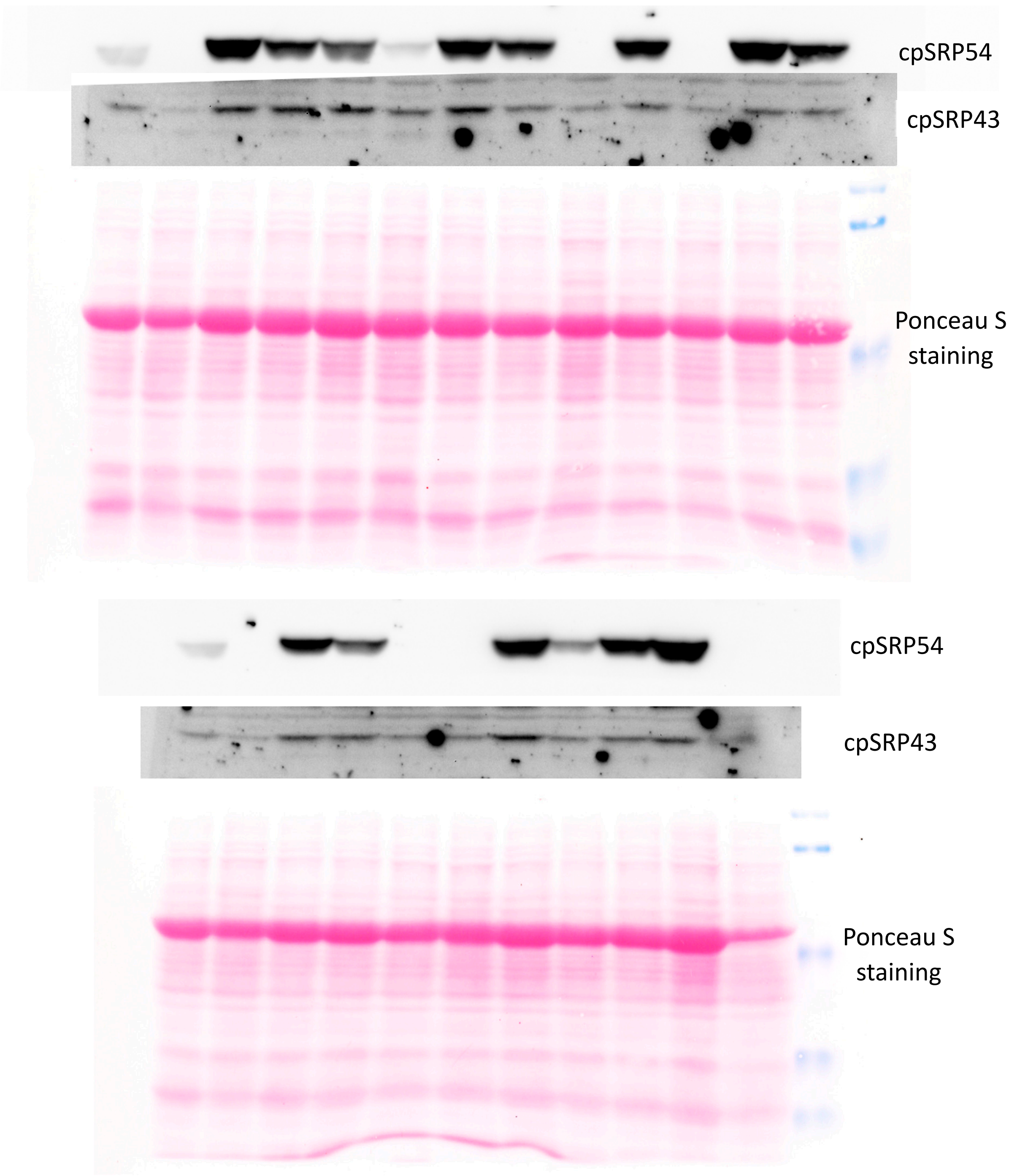

Figure 1

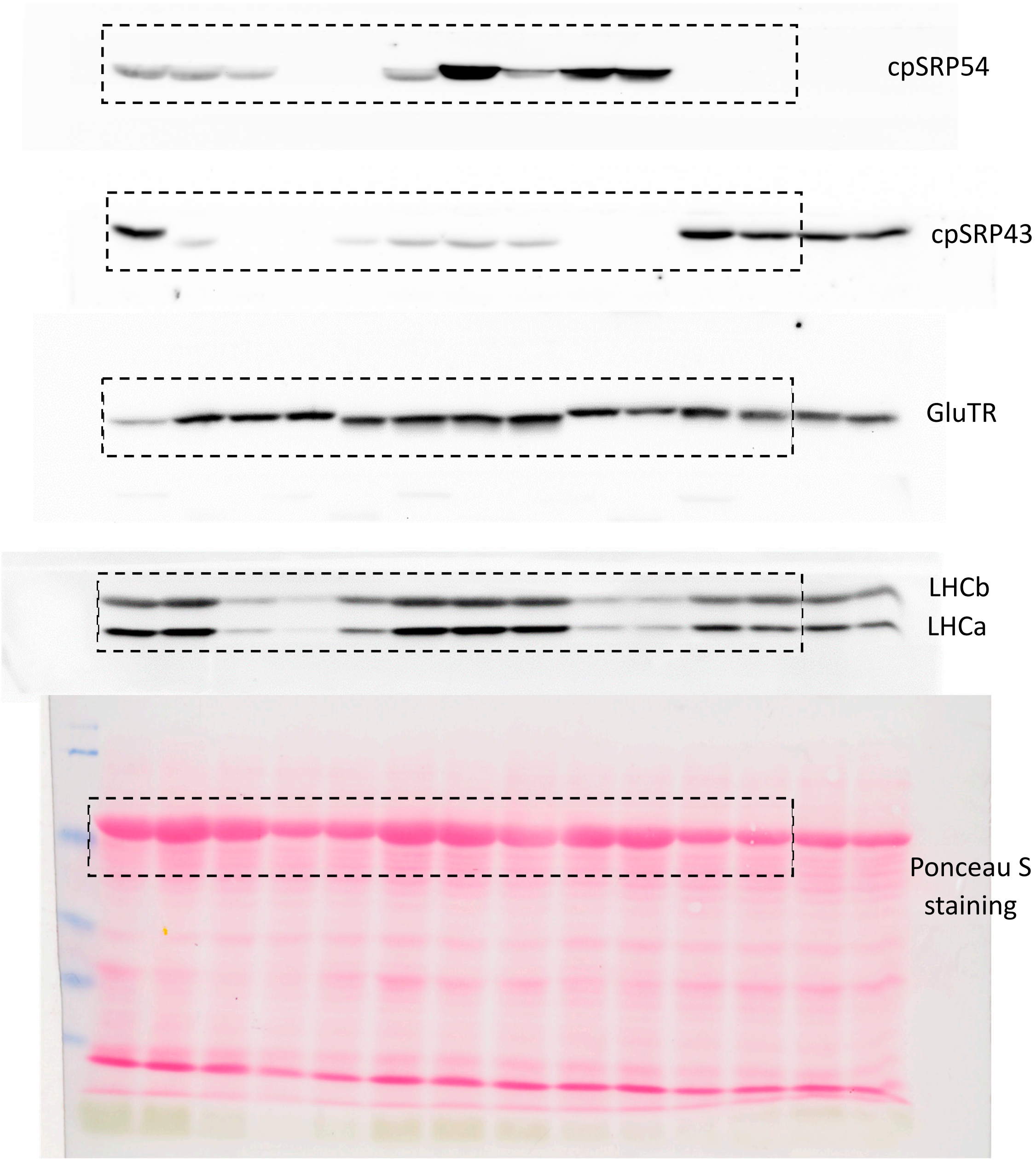

Figure 2

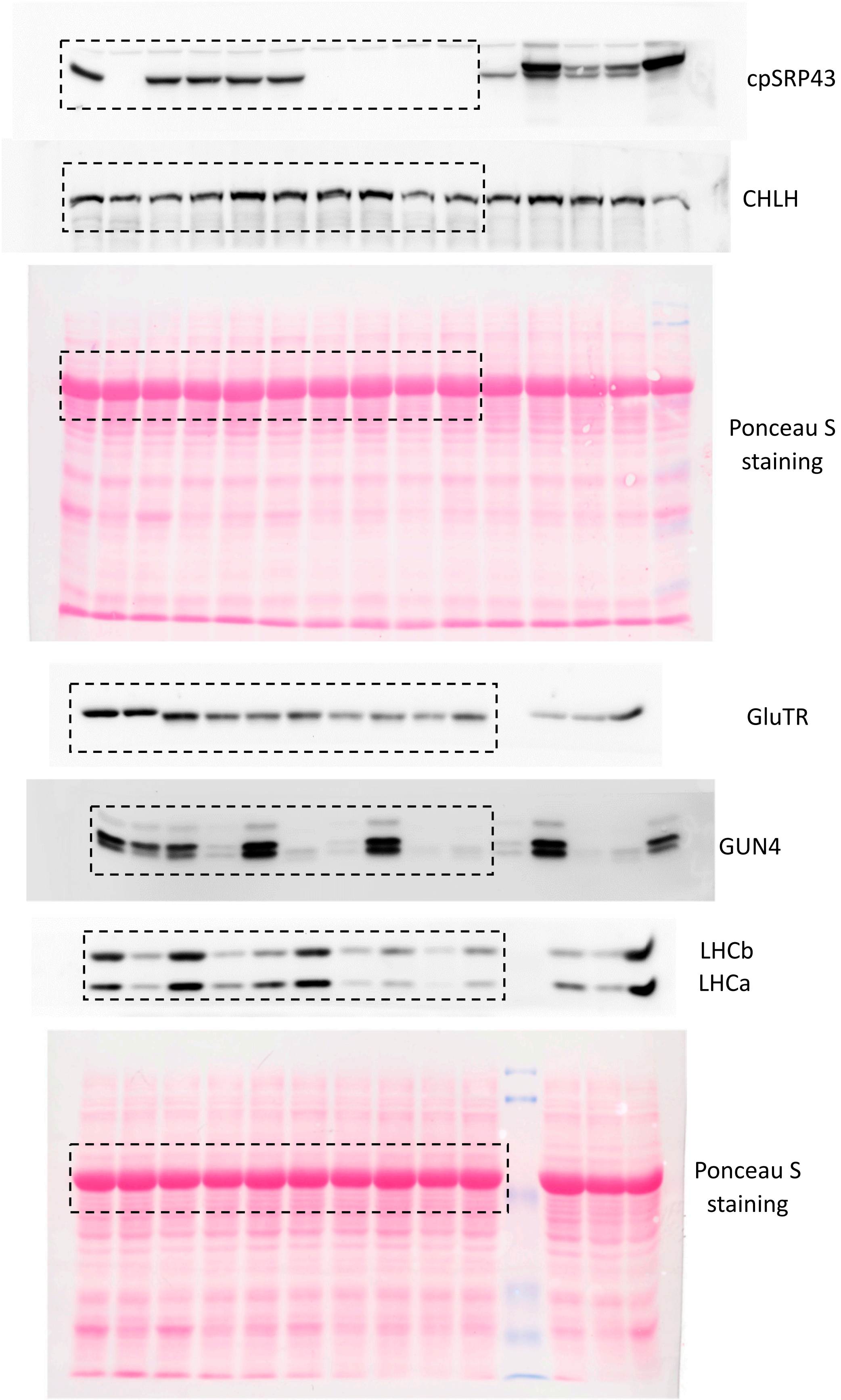
cpSRP43
CHLH
Ponceau S staining
GluTR
GUN4
LHCb
LHCa
Ponceau S staining

Figure 4

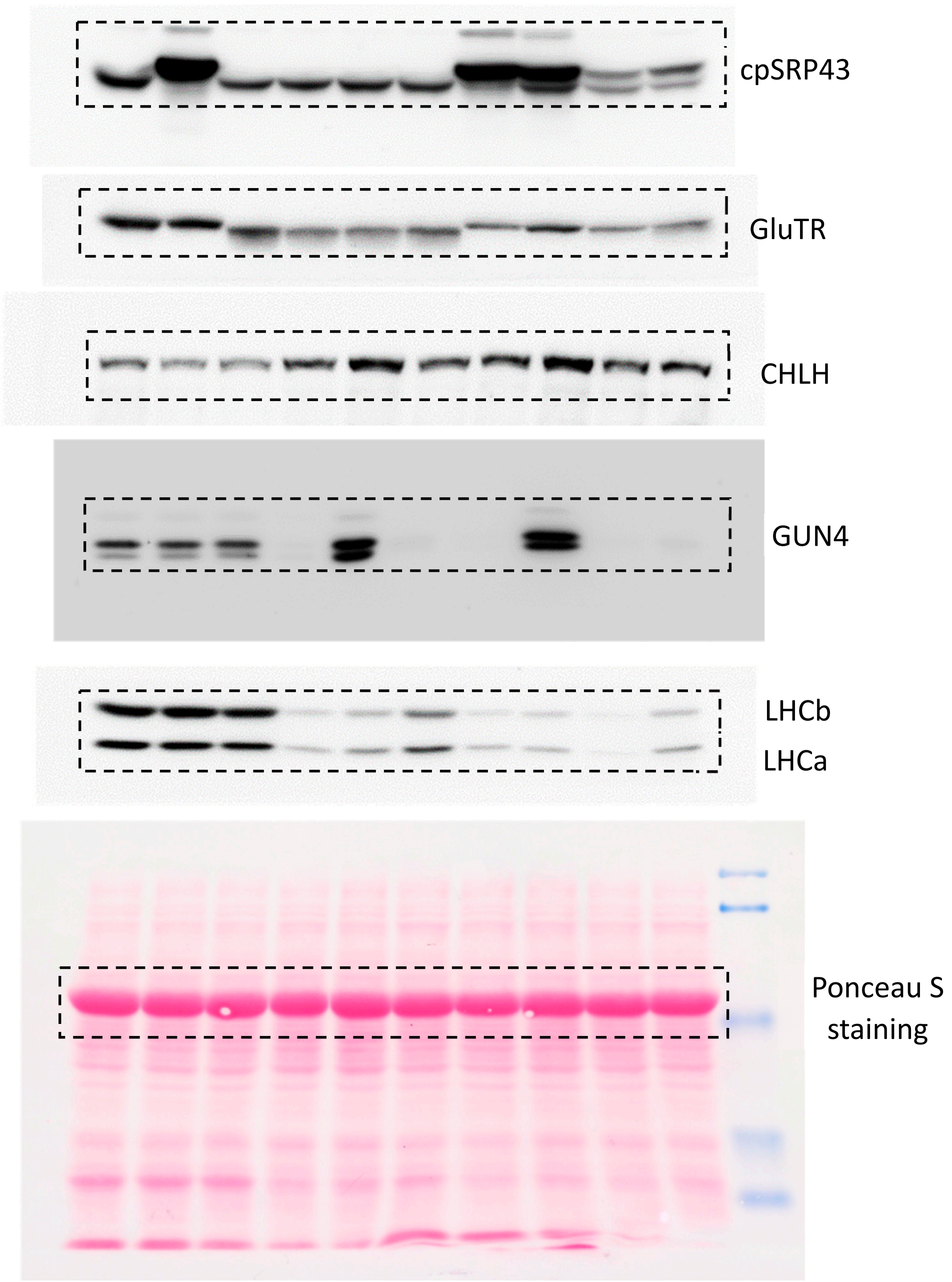

Figure 5

Relative protein levels	Lines	cpSRP54	cpSRP43
Col-0		1	1
ffc		0.097194	0.301107
cpSRP54-OX	*-1*	17.6588	5.677037
	-2	11.95903	6.855393
	-3	9.364544	5.946678
	-4	0.890893	2.936479
	-5	14.761	7.429173
	-6	12.43995	2.180247
	-7	0.10098	1.165242
	-8	13.88692	3.03501
	-9	0.120278	0.763281
	-10	17.84792	3.43638
	-11	13.34057	2.222148
	-12	12.47237	5.553105
	-13	7.641277	5.366705
	-14	0.243892	1.876642
	-15	0.234395	2.47341
	-16	15.3333	10.70303
	-17	3.731805	2.259107
	-18	13.28246	4.440848
	-19	19.35178	6.615205
	-20	0.368221	3.779551

Raw data for Figure 1C

Relative protein levels	cpSRP54	cpSRP43
cpSRP43-OX	1.17173	10.16455
Ler-0	1	1
chaos	0.57555	0.009568
chaos/ffc	0.009489	0.015226
ffc	0.013545	0.32003
Col-0	1	1
cpSRP54-OX-10	4.49963	1.267808
cpSRP54-OX-13	1.04042	0.902254

Raw data for Figure 2D

	MgP (pmol/mg)			MgMME (pmol/mg)			Pchlide (pmol/mg)			Chl *a* (nmol/mg)			Chl *b* (nmol/mg)			Heme (pmol/mg)		
cpSRP43-OX	0.028274	0.02913	0.036409	0.114121	0.115264	0.15432	1.508053	1.467696	1.73574	0.981854	0.980147	1.078694	0.23996	0.24201	0.261805	12.98206	11.95498	10.487356
Ler-0	0.034972	0.029896	0.03118	0.156815	0.142164	0.155233	1.85439	1.864737	1.935657	1.108801	1.075604	1.110832	0.260472	0.25652	0.264248	11.41463	10.93461	12.33785
chaos	0.028576	0.026387	0.02701	0.109269	0.083113	0.086338	0.525087	0.513433	0.516947	0.63156	0.597399	0.64559	0.098947	0.096715	0.102087	7.948183	12.21718	11.979472
ffc/chaos	0.006612	0.005635	0.00677	0.015844	0.015327	0.016525	0.094436	0.098155	0.115503	0.282327	0.255445	0.287895	0.05746	0.054631	0.062056	3.017114	1.910138	2.7722
ffc	0.015749	0.014637	0.013325	0.045247	0.044641	0.038463	0.325097	0.254878	0.293302	0.435287	0.432373	0.452917	0.107778	0.109114	0.113677	2.620939	3.075914	3.200271
Col-0	0.020397	0.024829	0.028656	0.078715	0.093586	0.139634	2.271424	2.360525	2.549869	0.911798	0.956392	0.933397	0.225692	0.235597	0.228517	10.1168	11.76381	11.76422
cpSRP54-OX-10	0.039563	0.046495	0.034935	0.22083	0.255892	0.15364	1.547632	1.878953	1.702129	0.913573	1.063419	0.975286	0.224949	0.255805	0.24146	12.68099	13.69751	12.35491
cpSRP54-OX-13	0.02178	0.020907	0.019807	0.09928	0.0773	0.075445	1.552203	1.487186	1.385893	1.005793	0.934753	0.946069	0.231375	0.222056	0.22752	6.164521	9.165173	11.31171
cpSRP43-OX/ffc-6	0.003474	0.008	0.005429	0.010459	0.018922	0.014539	0.086888	0.213279	0.15195	0.402273	0.478618	0.48943	0.110048	0.124654	0.132546	1.984581	3.129107	2.331491
cpSRP54-OX/chaos-1	0.016419	0.018588	0.023488	0.049053	0.049784	0.073572	0.424956	0.42375	0.421832	0.53475	0.537962	0.582976	0.087894	0.086132	0.099532	4.168585	5.862665	6.083263
cpSRP54-OX/chaos-3	0.017973	0.023337	0.016197	0.056383	0.066277	0.047656	0.352362	0.371083	0.295254	0.591511	0.521548	0.523057	0.093432	0.083092	0.082181	4.143971	4.663897	4.45241

Raw data for Figure 3

	MgP (pmol/mg)			MgMME (pmol/mg)			Pchlide (pmol/mg)			Chl *a* (nmol/mg)			Chl *b* (nmol/mg)		
Ler-0	0.034407	0.044161	0.042278	0.117911	0.173884	0.156196	2.099549	2.557559	2.473628	1.088623	1.154337	1.174947	0.297509	0.326628	0.323079
chaos	0.02679	0.029698	0.018998	0.09225	0.101685	0.069502	0.515173	0.607033	0.469792	0.791139	0.770932	0.77279	0.145534	0.141719	0.141706
Col-0	0.021544	0.019665	0.031347	0.096775	0.08148	0.127814	2.786539	2.16179	3.012893	1.203704	1.080729	1.108726	0.331384	0.3125	0.316664
gun4-1	0.024019	0.02173	0.026193	0.154448	0.139111	0.139297	0.396886	0.48318	0.446092	0.523402	0.537424	0.555818	0.055407	0.058879	0.060938
gun5-1	0.027726	0.027906	0.030666	0.191366	0.21479	0.186021	0.718232	0.697414	0.995974	0.684695	0.71223	0.821716	0.083123	0.087088	0.112361
gun4-3	0.033142	0.032998	0.026091	0.186466	0.170246	0.154025	1.700216	1.587331	1.8098	0.912267	0.866358	0.922681	0.178937	0.168847	0.18224
gun4-1/chaos	0.012379	0.014905	0.014788	0.090352	0.103575	0.092744	0.21535	0.24463	0.221381	0.376165	0.404717	0.367333	0.032939	0.036558	0.033271
gun5-1/chaos	0.042167	0.028378	0.029392	0.194614	0.155814	0.162326	0.677625	0.491506	0.581848	0.66818	0.567533	0.626253	0.084932	0.066128	0.076996
gun4-3/chaos-1	0.0114	0.012221	0.012044	0.084172	0.084789	0.09849	0.222251	0.300855	0.26716	0.38972	0.436293	0.419442	0.032209	0.039237	0.039546
gun4-3/chaos-2	0.029408	0.040989	0.034746	0.12849	0.138718	0.130233	0.622397	0.596305	0.609001	0.675917	0.667879	0.643535	0.098778	0.107597	0.097451

Raw data for Figures 4C–E

	MgP (pmol/mg)			MgMME (pmol/mg)			Pchlide (pmol/mg)			Chl *a* (nmol/mg)			Chl *b* (nmol/mg)		
Ler-0	0.007291	0.01197	0.032439	0.025143	0.052323	0.135451	2.033325	2.527732	2.375124	1.087345	1.153757	1.168353	0.275451	0.289034	0.293295
cpSRP43-OX	0.020335	0.021435	0.028451	0.085417	0.087249	0.128293	1.156766	2.290586	1.335495	1.221227	1.29269	1.324471	0.302876	0.324842	0.335497
Col-0	0.031598	0.03024	0.027529	0.137309	0.108967	0.09727	2.149534	2.209003	2.040323	1.233572	1.102431	1.123269	0.297428	0.287524	0.288672
gun4-1	0.026451	0.024314	0.028035	0.168123	0.155998	0.149744	0.200263	0.254784	0.208071	0.52518	0.585419	0.563516	0.055407	0.064367	0.059209
gun5-1	0.02853	0.028829	0.031601	0.187113	0.21479	0.194926	0.417465	0.212677	0.621994	0.716032	0.730216	0.816612	0.083123	0.084059	0.103151
gun4-3	0.033404	0.032586	0.025186	0.179534	0.168064	0.138862	0.734211	0.932804	1.295488	0.918907	0.892091	0.93503	0.167753	0.162075	0.17184
cpSRP43-OX/gun4-1	0.026674	0.018879	0.020238	0.115188	0.082524	0.089399	0.123005	0.050133	0.037937	0.526014	0.42247	0.454271	0.053356	0.046357	0.046807
cpSRP43-OX/gun5-1	0.034605	0.036889	0.034204	0.118261	0.166078	0.129857	0.221605	0.249839	0.230701	0.565967	0.661192	0.647013	0.066283	0.075704	0.074317
cpSRP43-OX/gun4-3-1	0.015993	0.01653	0.017457	0.081897	0.104697	0.095975	0.095765	0.10198	0.276946	0.584366	0.525391	0.564003	0.076229	0.057566	0.068488
cpSRP43-OX/gun4-3-2	0.042468	0.023677	0.036589	0.166362	0.070404	0.140401	0.408017	0.434423	0.52307	0.759253	0.706699	0.829554	0.12238	0.113519	0.119346

Raw data for Figures 5C–E

NF treatment	*RBCS*			*CP12*			*LHCB*			*PC*			*CA1*		
Ler-0	0.86300678	0.92494777	1.21204545	1.01831001	0.96337997	1.01831001	0.77793101	1.19558286	1.02648612	0.96980615	0.92387419	1.10631966	0.62370141	1.63458843	0.74171016
chaos	1.04785842	1.01216451	1.03343224	1.01127603	1.1298857	1.25368668	1.79964722	0.83376581	0.73596758	1.55376498	1.2190593	0.99707111	7.40719557	0.98549976	0.85792737
Col-0	0.91396416	1.01410673	1.07192911	0.96283608	1.14501151	0.89215241	0.80371397	1.12097497	1.07531106	0.80486026	1.16216123	1.03297851	0.87264763	1.19207179	0.93528057
gun4-1	2.21946122	1.64742124	2.29773035	3.44704971	2.93907485	4.18539012	10.7387492	6.65645504	10.9643932	7.60427251	4.71353759	8.91855656	61.5407929	31.6354701	154.7152

Raw data for Figure 6

DMSO treatment	*RBCS*			*CP12*			*LHCB*			*PC*			*CA1*		
Ler-0	1.1522837	0.9622582	0.88545811	1.27151011	0.69570275	1.03278714	1.26668573	1.00073406	0.73258021	1.3825116	0.84515718	0.77233122	1.16692533	1.07379028	0.75928439
chaos	1.03849621	1.17649563	1.67538883	0.94379337	0.85059421	1.00454552	1.11810729	1.05048724	1.05779395	1.06237388	1.11519159	0.84515718	0.93478873	1.19973207	0.87825528
Col-0	0.91307706	1.35548441	0.73143853	0.9088932	1.32150574	0.76960106	0.9713416	1.1471473	0.88151111	1.09243273	0.88120186	1.0263654	1.14992296	0.96028677	0.88979026
gun4-1	0.88810892	1.41304609	0.97861176	0.93444567	1.08086226	0.97412762	1.01259036	0.88151111	1.03386703	0.57736055	1.26360403	0.96429365	1.17408529	1.81697329	1.67195639

Raw data for Figure S1

References:

1. Klimyuk, V.I.; Persello-Cartieaux, F.; Havaux, M.; Contard-David, P.; Schuenemann, D.; Meiherhoff, K.; Gouet, P.; Jones, J.D.; Hoffman, N.E.; Nussaume, L. A chromodomain protein encoded by the Arabidopsis CAO gene is a plant-specific component of the chloroplast signal recognition particle pathway that is involved in LHCP targeting. *The Plant cell* **1999**, *11*, 87-99.
2. Wang, P.; Liang, F.C.; Wittmann, D.; Siegel, A.; Shan, S.O.; Grimm, B. Chloroplast SRP43 acts as a chaperone for glutamyl-tRNA reductase, the rate-limiting enzyme in tetrapyrrole biosynthesis. *Proceedings of the National Academy of Sciences of the United States of America* **2018**, *115*, E3588-E3596, doi:10.1073/pnas.1719645115.
3. Amin, P.; Sy, D.A.; Pilgrim, M.L.; Parry, D.H.; Nussaume, L.; Hoffman, N.E. Arabidopsis mutants lacking the 43-and 54-kilodalton subunits of the chloroplast signal recognition particle have distinct phenotypes. *Plant physiology* **1999**, *121*, 61-70.
4. Ji, S.; Grimm, B.; Wang, P. Chloroplast SRP43 and SRP54 independently promote thermostability and membrane binding of light ‐ dependent protochlorophyllide oxidoreductases. *The Plant Journal* **2023**, *115*, 1583-1598.
5. Larkin, R.M.; Alonso, J.M.; Ecker, J.R.; Chory, J. GUN4, a regulator of chlorophyll synthesis and intracellular signaling. *Science* **2003**, *299*, 902-906.
6. Mochizuki, N.; Brusslan, J.A.; Larkin, R.; Nagatani, A.; Chory, J. Arabidopsis genomes uncoupled 5 (GUN5) mutant reveals the involvement of Mg-chelatase H subunit in plastid-to-nucleus signal transduction. *Proceedings of the National Academy of sciences* **2001**, *98*, 2053-2058.
7. Woodson, J.D.; Perez-Ruiz, J.M.; Chory, J. Heme synthesis by plastid ferrochelatase I regulates nuclear gene expression in plants. *Current Biology* **2011**, *21*, 897-903.
